# 5-Aminosalicylic Acid Distribution into the Intestinal Membrane Along the Gastrointestinal Tract After Oral Administration in Rats

**DOI:** 10.3390/pharmaceutics16121567

**Published:** 2024-12-07

**Authors:** Yorinobu Maeda, Yuta Goto, Fumiya Ohnishi, Syoutarou Koga, Satoshi Kawano, Yuhzo Hieda, Takeshi Goromaru, Teruo Murakami

**Affiliations:** 1 Laboratory of Drug Information Analytics, Faculty of Pharmacy & Pharmaceutical Sciences, Fukuyama University, Hiroshima 729-0292, Japan; p7120040@fukuyama-u.ac.jp (Y.G.); p7120019@fukuyama-u.ac.jp (F.O.); p7120039@fukuyama-u.ac.jp (S.K.); p7119026@fukuyama-u.ac.jp (S.K.); goromaru@fukuyama-u.ac.jp (T.G.); 2 Faculty of Pharmacy and Pharmaceutical Sciences, Fukuyama University, 1 Sanzo, Fukuyama 729-0292, Japan; hieda@fukuyama-u.ac.jp; 3 Faculty of Pharmaceutical Sciences, Hiroshima International University, 5-1-1 Hiro-koshingai, Kure 737-0112, Japan

**Keywords:** 5-aminosalicylic acid, acetyl-5-aminosalicylic acid, poorly soluble drug, suspension, high-dose drug, pH-dependent solubility, membrane distribution, colonic delivery, rats

## Abstract

Background: 5-Aminosalicylic acid (5-ASA), the first-line therapy for ulcerative colitis, is a poorly soluble zwitterionic drug. Unformulated 5-ASA is thought to be extensively absorbed in the small intestine. Methods: The pH-dependent solubility of 5-ASA in vitro and the intestinal membrane distribution of 5-ASA and its N-acetyl metabolite (AC-5-ASA) after the oral administration of 5-ASA were examined in fed rats. 5-ASA was administered as a suspension in water, 0.1 M HCl, or 0.1 M NaOH to untreated rats or as a solution in 5% NaHCO_3_ to lansoprazole-pretreated rats. Results: 5-ASA solubility in vitro was higher at pH < 2 and pH > 7. In rats, the 5-ASA and AC-5-ASA were detected mostly in the small intestine at 3 h and in the colonic region at 8 h after administration. The dosing vehicle (suspension or solution) and lansoprazole pretreatment did not significantly affect the pH of the luminal fluid in rats or the 5-ASA distribution in membranes. Conclusions: The 5-ASA distribution in membranes in the proximal intestine was found to be restricted by the intrinsic regional luminal pH, low solubility, and saturable membrane permeability. Unabsorbed 5-ASA in the proximal intestine was delivered to the distal intestine. The higher the oral dose of 5-ASA, the more 5-ASA may be delivered to the distal intestine due to the restricted absorption in the small intestine.

## 1. Introduction

Inflammatory bowel diseases (IBDs) ulcerative colitis (UC) and Crohn’s disease (CD) have similar symptoms and lead to digestive disorders and inflammation in the digestive system. UC is colonic inflammation that affects the rectum only or progresses proximally to the involved part of or the entire colon. In contrast, CD affects the entire gastrointestinal tract from mouth to anus, in which the distal parts of the small intestine or ileum and colon are commonly affected. UC and CD prevalences are increasing worldwide [1,2,3]. 5-Aminosalicylic acid (5-ASA), also called mesalamine and mesalazine, is the first line of therapy for the remission induction and maintenance of mild-to-moderate UC. Various chemically modified derivatives and pharmaceutically modified colon-targeting delivery systems for 5-ASA have been developed and are clinically available [4,5]. 5-ASA has a variety of pharmacological effects, making this class of agents pluripotent from an anti-inflammatory standpoint. These effects include interference in the metabolism of arachidonic acid to prostaglandins and leukotrienes, the scavenging of reactive oxygen species, cyclo-oxygenase inhibition, leucocyte function, tumor necrosis factor activation, and producing the cytokines lipoxygenase, platelet-activating factor, and interleukin-1, as well as nuclear factor κB, B cells, and oxygen radicals [6,7]. Furthermore, 5-ASA can inhibit peptide transporter 1 (PEPT1)-mediated uptake transport, where PEPT1-mediated transport is upregulated under inflamed conditions and PEPT1 transports antigenic proinflammatory oligopeptides, such as formyl, methionine, leucine, and phenylalanine [8].

It is generally accepted that orally administered unformulated 5-ASA is extensively absorbed in the proximal small intestine and specific delivery systems are required to deliver 5-ASA to the UC-affected colon. In rats, it has been reported that 91% of 5-ASA was absorbed for 24 h in the proximal small intestine when rats received 20 mg 5-ASA via a duodenostomy tube [9]. Similarly, in humans, it has been reported that only 0.1% of the total 5-ASA dose was recovered in the duodenal aspirate when human volunteers received 150 mg pure 5-ASA in a 100 mL pH 7.0 buffer solution as an installation into a 100 cm long proximal small bowel section for 6 h [10]. These data indicate that unformulated 5-ASA is extensively absorbed and metabolized into N-acetyl-5-ASA (AC-5-ASA) in the upper gastrointestinal tract [9,10,11]. 5-ASA’s reported oral bioavailability is low due to the extensive first-pass metabolism, where only 5.79 ± 0.24% of intact 5-ASA was transferred into the portal circulation in an in situ rat small intestinal perfusion study [12], and 5-ASA’s absolute oral bioavailability was 15.5%, in which the intravenous 5-ASA dose was 0.50 g and the oral dose was 2.42 g in healthy volunteers [13].

However, in the Biopharmaceutics Classification System (BCS), 5-ASA is classified as a BCS Class IV drug with low solubility and permeability [12,14,15,16]. In clinical settings, high-dose 5-ASA therapy is also conducted for adult patients with moderately active disease [17,18,19]. In addition, the 5-ASA membrane transport is saturable due to carrier-mediated transepithelial transport at low doses and dominated by the passive, paracellular process in higher doses in situ rat intestinal perfusion and Caco-2 cells [12,15,16,20]. The influx transporters for 5-ASA in gastrointestinal absorption are organic anion-transporting polypeptide 2B1 (OATP2B1) and sodium-coupled monocarboxylate transporter 1 (SMCT1), with a greater contribution of OATP2B1 than SMCT1 [21]. AC-5-ASA, but not 5-ASA, is a substrate for an ATP-dependent efflux transporter called multidrug resistance-associated protein 2 (MRP2) [22,23,24]. In the present study, we examined the pH-dependent 5-ASA solubility in vitro and the intestinal membrane distribution of 5-ASA and AC-5-ASA along the gastrointestinal tract 3 and 8 h after the 5-ASA oral administration at a 30 mg/2.2 mL/kg dose in fed rats. In the rat study, 5-ASA was administered as a suspension in water, 0.1 M HCl, or 0.1 M NaOH to untreated control-fed rats, and as a solution of 5-ASA in 5% NaHCO_3_ to lansoprazole-pretreated fed rats. Pretreatment with lansoprazole, a proton pump inhibitor, was undertaken to modify the luminal fluid pH. The data obtained in these studies would clarify whether unformulated 5-ASA is mostly absorbed from the small intestine, or 5-ASA is delivered to the colonic region when 5-ASA is administered at a high dose. In addition, the results may be useful as reference data in evaluating the usefulness of newly developed colonic delivery systems of 5-ASA.

## 2. Materials and Methods 

### 2.1. Materials 

5-ASA and 4-aminosalicylic acid (4-ASA), an internal standard (I.S.) in high-performance chromatography (HPLC) analysis, were purchased from FUJIFILM Wako Chemical Co., Japan. Lansoprazole was a gift from Takeda Chemical Industries Ltd., Osaka, Japan. All reagents and solvents were of reagent grade and purchased from FUJIFILM Wako Chemical Co., Osaka, Japan.

### 2.2. Synthesis of AC-5-ASA, N-Propionyl-5-ASA (Prop-5-ASA), and N-Propionyl-4-ASA (Prop-4-ASA)

AC-5-ASA, the main 5-ASA metabolite, was synthesized by the reaction of 5-ASA with acetic anhydride. Briefly, 1000 mg (6.54 mmol) of 5-ASA was dissolved in 30 mL of dichloromethane and 30 mL of ethanol, and 876 μL (8.58 mmol) of acetic anhydride was added. The solution was stirred at 50 °C for 1 h, and then evaporated. Prop-5-ASA and Prop-4-ASA were synthesized in the same manner as AC-5-ASA using 1116 μL (8.58 mmol) of propionic anhydride instead of acetic anhydride. The obtained crude AC-5-ASA, Prop-5-ASA, and Prop-4-ASA were recrystallized from ice water (88, 84, and 98% yields, respectively). To examine the recrystallized AC-5-ASA’s purity, thin-layer chromatography (TLC) was carried out on precoated silica gel plates (Kieselgel 60 F_254_ plate, Merck, Darmstadt, Germany) using chloroform: methanol: acetic acid: distilled water (15:5:2:1, *v*/*v*/*v*/*v*) as the developing solvent. Spot detection was undertaken using short-wave ultraviolet light (254 nm). The Rf values of 4-ASA, 5-ASA, AC-5-ASA, Prop-5-ASA, and Prop-4-ASA were 0.42, 0.41, 0.73, 0.82, and 0.93, respectively. The synthesized compounds’ purity was also examined by recording the proton nuclear magnetic resonance (^1^H-NMR) spectra on a JEOL JNM-400S at 400 MHz (JEOL Ltd., Tokyo, Japan), and the chemical shifts relative to tetramethylsilane (TMS, an internal standard, δ 0.00) were estimated. The NMR spectra were measured in DMSO-d6 (δ 2.49). 

### 2.3. 5-ASA Solubility

Tris-HCl buffer (0.1 M) solutions with different pHs (2.0, 4.0, 6.0, 6.5, 7.0, 7.5, and 8.0) were used. 5-ASA (approximately 100 mg) was suspended in each buffer solution (about 1 mL). The suspension was shaken vigorously for 30 min and/or 12 h at 25 °C. Separately, 5-ASA solubilities in pure water, 0.1 M HCl solution, 0.1 M NaOH solution, and 5% NaHCO_3_ solution were also determined (30 min of shaking at 25 °C) because these vehicles were used to administer 5-ASA in the rat studies. After shaking, the suspension was centrifuged at 3000× *g* for 10 min, and the supernatant was filtrated through a syringe filter with a 0.22 µm pore size (Millipore, Tokyo, Japan). HPLC was used to measure the 5-ASA concentration in each filtrate. 

### 2.4. 5-ASA Membrane Distribution Study in Rats 

The animal study was performed according to the Care and Use of Laboratory Animals of the Committee for Animal Experiments of Fukuyama University (2023-A-10). Male Sprague Dawley rats were purchased from Japan SLC, Inc. (Hamamatsu, Japan). After feeding for several days, the rats that weighed 200 to 300 g were used for the membrane distribution studies. The fed rats were used without fasting, and each 5-ASA suspension in water, 0.1 M HCl solution, or 0.1 M NaOH solution was administered orally at a 30 mg 5-ASA/2.2 mL/kg dose. Separately, the fed rats were treated with lansoprazole, which was dissolved with a small amount of dimethyl sulfoxide (DMSO) and then diluted with physiological saline to make a lansoprazole solution (5 mg/mL); this solution was injected intraperitoneally at a 5 mg/mL/kg dose. One hour later, the 5-ASA solution dissolved in 5% NaHCO_3_ was administered orally at a 30 mg 5-ASA/2.2 mL/kg dose to the lansoprazole-treated rats. At 3 or 8 h after the oral administration of the 5-ASA suspension or solution, the rats were sacrificed using a pentobarbital overdose and a laparotomy was performed to remove the stomach, small intestine (divided into 4 portions with equal length), cecum, and colon (divided into 3 portions with equal length). One milliliter of water was poured into each isolated segment’s lumen and the luminal fluid pH was measured using a compact pH meter (HORIBA, LAQUA twin^®^, Kyoto, Japan). The intestinal tract was opened and cleaned with ice-cold 0.9% NaCl solution, frozen in liquid nitrogen, and stored at −80 °C until analysis. Ten percent of the intestinal membrane homogenates in ice-cold 0.9% NaCl solution were prepared to measure the 5-ASA and AC-5-ASA concentrations in the membrane using a Polytron^®^ homogenizer (PT1200E, Kinematica AG, Malters, Switzerland). Separately, from some rats, blood was collected at 0.5, 3, or 8 h using a heart puncture, and urine in the bladder was collected at 0.5, 3, or 8 h after the 5-ASA oral administration.

### 2.5. Analysis of 5-ASA and AC-5-ASA Using HPLC

The 5-ASA and AC-5-ASA concentrations in the samples were determined using HPLC and 4-ASA as the I.S. using a reported method with extensive modifications [25]. An L-column 2 ODS (150 mm × 4.6 mm, particle size 5 µm, CERI Co., Ltd., Saitama, Japan) was used, and the mobile phase was a mixture of 0.1 M acetic acid, acetonitrile, and triethylamine (920:100:2, *v*/*v*/*v*). The mobile phase flow rate was 1 mL/min. Detection was achieved using a fluorescence detector at 315 and 430 nm for the excitation and emission wavelengths, respectively. Each 5-ASA-containing sample (100 µL) was mixed with 900 µL of methanol for deproteinization. The mixture was vigorously mixed by a vortex mixer for 30 s and centrifuged at 15,000× *g* for 5 min at 4 °C, and all the supernatant was collected and evaporated. Then, 100 µL of I.S. solution (5 µg of 4-ASA/mL), 900 µL of 0.05 M phosphate buffer (pH 7.4), and 100 µL of propionic anhydride were added, and the reaction solutions were incubated at 37 °C for 1 h. The compounds of interest were extracted with 4 mL of acetonitrile after adding 1 mL of 10% sodium chloride solution. Liquid phases were separated by centrifugation at 3000× *g* for 5 min after cooling at 4 °C. The organic layer was removed and evaporated to dryness under air. The residue was dissolved in 250 µL of mobile phase, and 10 µL aliquots were injected into the HPLC column. The HPLC system (Shimadzu, Kyoto, Japan) comprised a model LC20AD pump, a 20 µL fixed injection loop, and a fluorescence spectrophotometer (Shimadzu, RF10AXL). Data acquisition was performed with the Sept 3000 processor (Hangzhou, China). The retention times for the AC-5-ASA, Prop-5-ASA, and Prop-4-ASA (I.S.) were 4.8, 8.7, and 10.6 min, respectively. The correlation coefficient (r) values of the calibration curves for 5-ASA and AC-5-ASA were more than 0.9997 and 0.9999, respectively, with a low Y-intercept value and variation within 5% in a concentration range from 1 to 10 µg/mL for both. The 5-ASA and AC-5-ASA detection limits were 100 and 50 ng/mL (or ng/g), respectively.

### 2.6. Statistical Analysis

The measurement data were expressed as the mean ± standard deviation (SD). Statistical analysis was performed using the unpaired Student’s *t*-test. *p*-values less than 0.05 were statistically significant.

## 3. Results

### 3.1. Synthesis of N-Acetyl-5-ASA (AC-5-ASA), N-Propionyl-5-ASA (Prop-5-ASA), and N-Propionyl-4-ASA (Prop-4-ASA)

The chemical shifts measured in DMSO-d6 (δ 2.49) relative to tetramethylsilane (TMS δ 0.00) were as follows: AC-5-ASA: ^1^H NMR (400 MHz, DMSO-d6) δ: 2.02 (s, 3H), 6.91 (d, *J* = 8.7 Hz, 1H), 7.65 (dd, *J* = 8.7, 2.7 Hz, 1H), 8.09 (d, *J* = 2.7 Hz, 1H), 9.91 (s, 1H). Prop-5-ASA: ^1^H NMR (400 MHz, DMSO-d6) δ: 1.08 (t, *J* = 7.5 Hz, 3H), 2.29 (q, *J* = 7.5 Hz, 2H), 6.91 (d, *J* = 8.7 Hz, 1H), 7.66 (dd, *J* = 8.7, 2.7 Hz, 1H), 8.12 (d, *J* = 2.7 Hz, 1H), 9.83 (s, 1H). Prop-4-ASA: ^1^H NMR (400 MHz, DMSO-d6) δ: 1.05 (t, *J* = 7.5 Hz, 3H), 2.33 (q, *J* = 7.5 Hz, 2H), 7.04 (dd, *J* = 8.7, 2.3 Hz, 1H), 7.34 (t, *J* = 2.3 Hz, 1H), 7.68 (d, *J* = 8.7 Hz, 1H), 10.12 (s, 1H). The compounds synthesized in this study were all chromatography pure according to NMR and HPLC spectra. The ^1^H-NMR spectra of AC-5-ASA, Prop-5-ASA, and Prop-4-ASA are shown in Figure S1A–C in the Supplementary Materials.

### 3.2. 5-ASA Solubility

**Figure 1:**
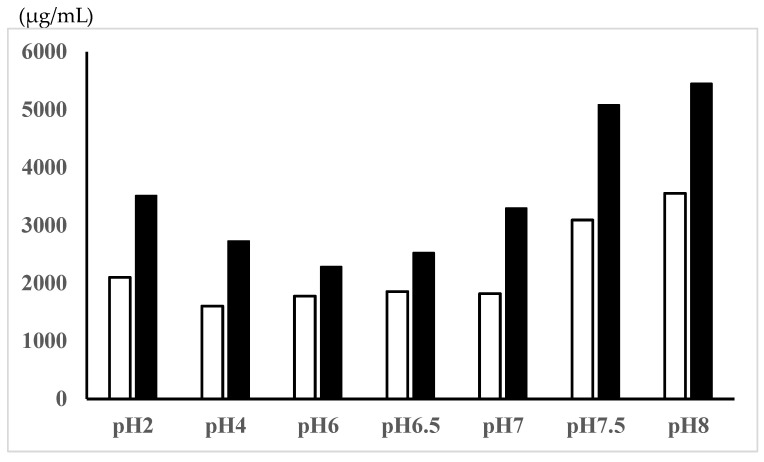
5-ASA solubility in Tris-HCl buffers with different pHs. 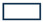 30 min incubation, 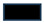 12 h incubation with shaking.

The solubilities of 5-ASA, a zwitterionic compound, in the Tris-HCl buffer solutions with different pHs are shown in Figure 1. The 5-ASA solubility was higher at pH < 2 (most positively charged) and pH > 7 (most negatively charged) compared with the zwitterionic pH range (pH 4, 5, 6.5). In the rat study, 5-ASA was administered as a suspension (30 mg of 5-ASA in 2.2 mL of water, 0.1 M HCl, or 0.1 M NaOH/kg) or as a solution (30 mg of 5-ASA in 2.2 mL of 5% NaHCO_3_/kg). The 5-ASA solubilities in these dosing vehicles were determined to be in the following order: 5% NaHCO_3_ > 0.1 M NaOH > 0.1 M HCl > water (Table 1).

### 3.3. 5-ASA Membrane Distribution After Oral Administration in Rats

#### 3.3.1. Comparison of 5-ASA Membrane Distribution Between 5-ASA Suspension in Water in Untreated Control Rats and 5-ASA Solution in 5% NaHCO_3_ in Lansoprazole-Pretreated Rats

To examine the solubility (or pH) effects of 5-ASA in the dosing vehicle on the 5-ASA membrane distribution, 5-ASA was administered as a suspension in water (11.7% dissolved and 88.8% solid) to untreated control rats or as a solution in 5% NaHCO_3_ to lansoprazole-treated rats at a 30 mg 5-ASA/2.2 mL/kg dose. Then, the 5-ASA and AC-5-ASA plasma concentrations at 0.5, 3, and 8 h, the 5-ASA and AC-5-ASA urine concentrations in the bladder at 0.5, 3, and 8 h, and the 5-ASA and AC-5-ASA membrane concentrations in the small and large intestines at 3 and 8 h after the oral administration were compared (Table 2). The 5-ASA was rapidly absorbed after the dosing, as shown by the plasma and urine concentrations evaluated at 0.5 h after the administration. There was no significant difference in the 5-ASA and AC-5-ASA concentrations in the plasma and urine between the 5-ASA suspension in water and the 5% NaHCO_3_ solution. In addition, there was no significant difference in the 5-ASA and AC-5-ASA membrane concentrations between the 5-ASA suspension and the 5-ASA solution. These results indicate that the 5-ASA solubility in the dosing preparations and lansoprazole treatment did not significantly affect the extent of the intestinal membrane distribution of 5-ASA in the fed rats with food in their stomach. 

#### 3.3.2. Comparison of 5-ASA Regional Membrane Distribution Between 5-ASA Suspension in Water and 5-ASA Solution in 5% NaHCO_3_ 3 h After Administration

**Figure 2:**
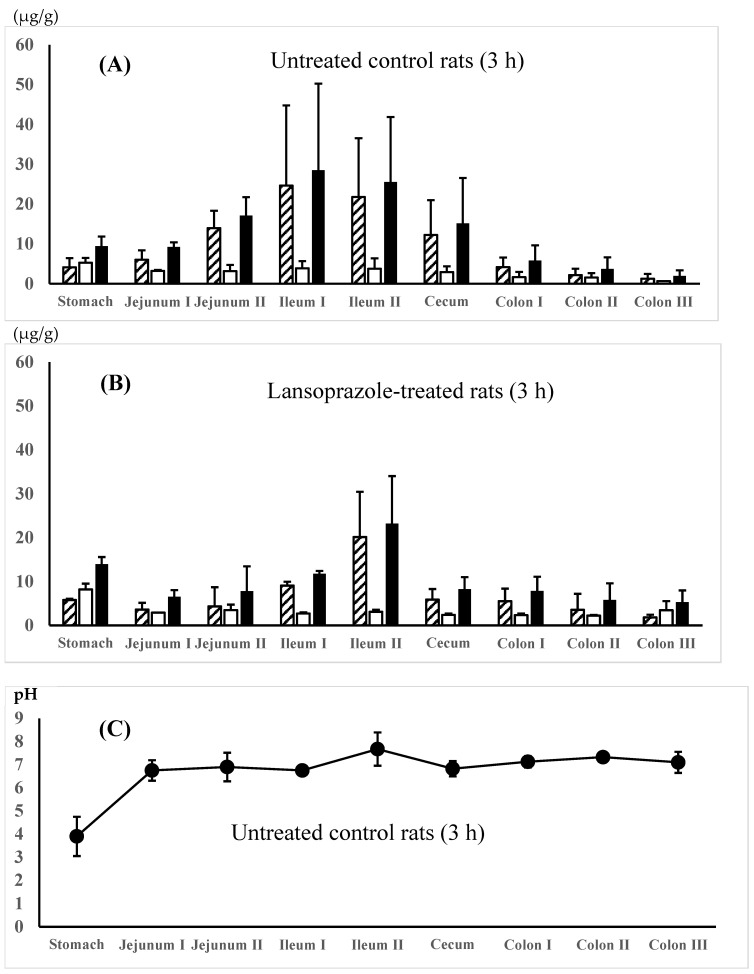

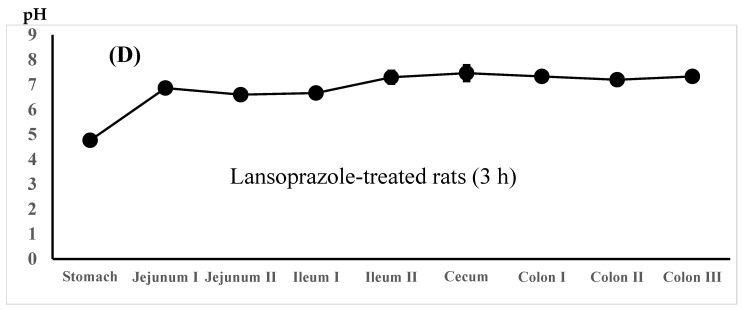
5-ASA and AC-5-ASA concentrations in the intestinal membranes 3 h after the oral administration of the 5-ASA suspension in water to the untreated control rats (**A**) and the 5-ASA solution in 5% NaHCO_3_ to the lansoprazole-pretreated rats (**B**) at a 30 mg/2.2 mL/kg dose. Concn. of 5-ASA, 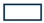; concn. of AC-5-ASA, 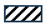; total (5-ASA + AC-5-ASA) concn., 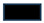. Regional luminal fluid pH along the GI tract 3 h after the administration of the 5-ASA suspension in water to the untreated control rats (**C**) and the 5-ASA solution in 5% NaHCO_3_ to the lansoprazole-pretreated rats (**D**) at a 30 mg/2.2 mL/kg dose. Each value represents the mean ± S.D. of 3 trials. There was no significant difference in each corresponding value between the untreated control and the lansoprazole-treated rats.

The regional membrane distributions of 5-ASA and AC-5-ASA along the gastrointestinal tract were compared between the 5-ASA suspension in water (dissolved fraction 11.7%, solid fraction 88.3%) in the untreated control rats and the 5-ASA solution in 5% NaHCO_3_ in the lansoprazole-pretreated rats at 3 h after the 5-ASA oral administration. Figure 2A,B show that higher total (5-ASA + AC-5-ASA) concentrations were observed in the small intestine, especially the ileum region, compared with the other regions. When the maximal total concentrations of 5-ASA and AC-5-ASA were compared between the small intestinal and the colonic regions, the small intestinal region showed higher concentrations than the colonic region by 5.5-fold for water suspension (Figure 2A) and 2.8-fold for 5% NaHCO_3_ solution (Figure 2B). A micro-pH sensor determined the regional luminal fluid pHs. The gastric pHs of untreated control rats and lansoprazole-pretreated rats were 3.90 ± 0.60 and 4.77 ± 0.12, respectively, indicating that lansoprazole pretreatment increased gastric pH even 3 h after pretreatment, although a significant difference was not detected between them (*p* = 0.07) (Figure 2C,D). Taken together, there was no significant difference in the regional fluid pH and 5-ASA membrane distribution between the 5-ASA suspension in the untreated control rats and the 5-ASA solution in the lansoprazole-pretreated rats.

#### 3.3.3. Comparison of Regional Membrane Distribution of 5-ASA Between 5-ASA Suspension in Water, 0.1 M HCl, or 0.1 M NaOH in Untreated Control Rats and 5-ASA Solution in 5% NaHCO_3_ in Lansoprazole-Pretreated Rats 8 h After Administration

**Figure 3:**
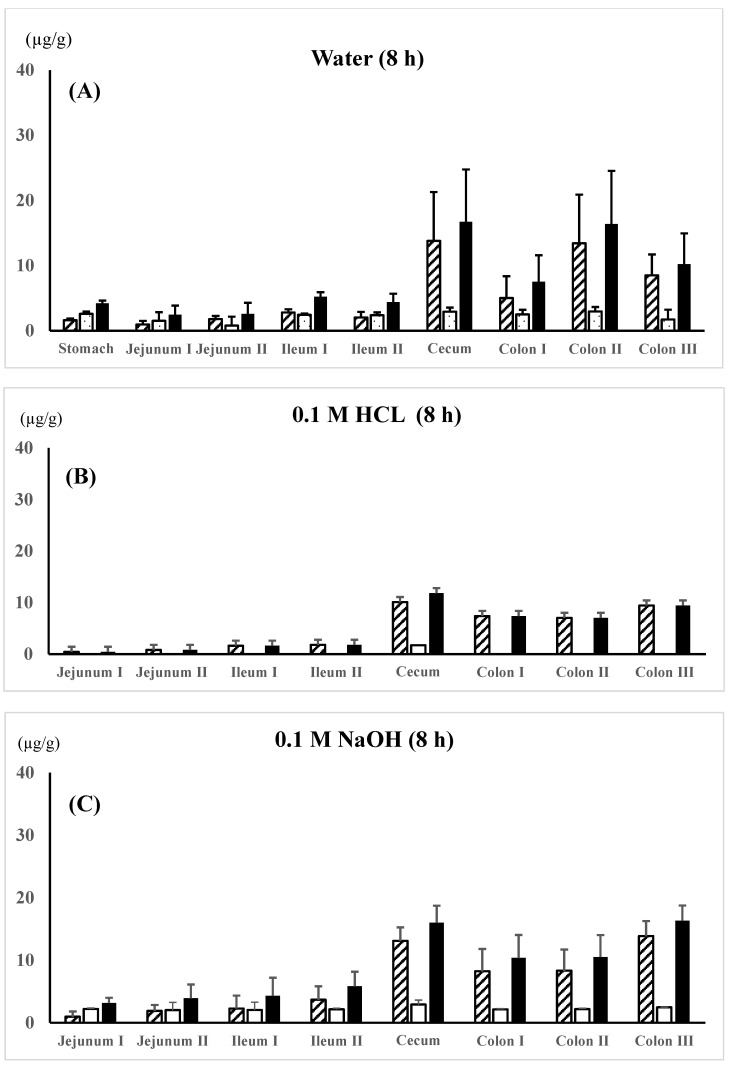

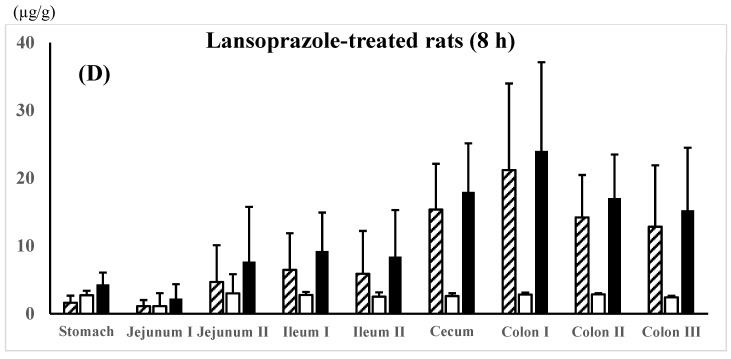
5-ASA and AC-5-ASA concentrations in the intestinal membranes 8 h after the oral administration of the 5-ASA suspension in water (**A**), 0.1 M HCl (**B**), or 0.1 M NaOH (**C**) to the untreated control rats and the 5-ASA solution in 5% NaHCO_3_ to the lansoprazole-pretreated rats (**D**) at a 30 mg/2.2 mL/kg dose. 5-ASA concn., 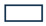; AC-5-ASA concn., 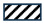; total (5-ASA + AC-5-ASA) concn., 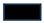. Each value represents the mean ± S.D. of three trials. There was no significant difference in each corresponding value between the different dosing preparations.

5-ASA and AC-5-ASA regional membrane distributions along the gastrointestinal tract were compared between the 5-ASA suspension in water, 0.1 M HCl, or 0.1 M NaOH in the untreated control rats and the 5-ASA solution in 5% NaHCO_3_ in the lansoprazole-pretreated rats at 8 h after the 5-ASA oral administration (Figure 3A–D). In all dosing preparations, the average total concentrations of 5-ASA and AC-5-ASA in the colonic region were higher than those in the small intestinal region; for example, as follows: colon II > colon III > colon I > ileum I > ileum II > jejunum I, II for suspension in water (Figure 3A) and colon I > colon II > colon III > ileum I > ileum II > jejunum II > jejunum I for solution in 5% NaHCO_3_ (Figure 3D). When the maximal total concentrations of 5-ASA and AC-5-ASA were compared between the small intestinal and the colonic regions, the colonic region showed higher concentrations than the small intestinal region by 3.0-fold for water suspension (Figure 3A), 5.2-fold for 0.1M HCl (Figure 3B), 2.8-fold for 0.1M NaOH (Figure 3C), and 2.2-fold for 5% NaHCO_3_ (Figure 3D) at 8 h after administration. These results indicate that 5-ASA administered at a higher dose is distributed into the colonic membranes and not just the small intestine. No significant difference in the 5-ASA and AC-5-ASA membrane concentrations was observed between the different dosing preparations (water, 0.1 M HCl, 0.1 M NaOH, and 5% NaHCO_3_). 

**Figure 4:**
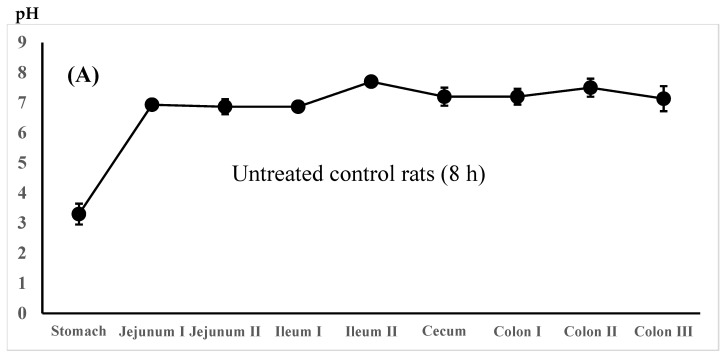

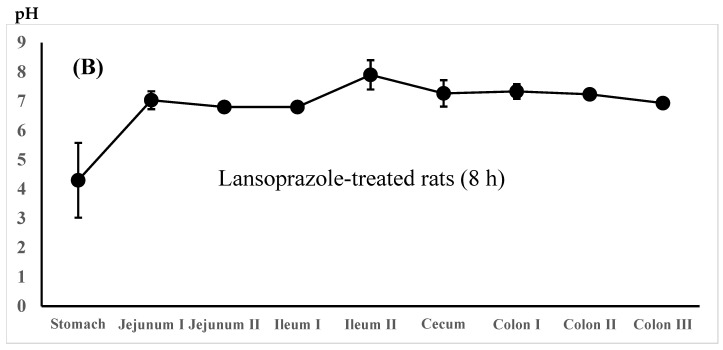
Regional luminal fluid pH along the GI tract 8 h after the administration of the 5-ASA suspension in water to the untreated control rats (**A**) and the 5-ASA solution in 5% NaHCO_3_ to the lansoprazole-pretreated rats (**B**) at a 30 mg/2.2 mL/kg dose. Each value represents the mean ± S.D. of three trials. There was no significant difference in each corresponding value between the untreated control and the lansoprazole-treated rats.

The regional luminal fluid pH along the GI tract was also determined in the untreated control rats administered the 5-ASA suspension in water and the lansoprazole-pretreated rats administered the 5-ASA solution in 5% NaHCO_3_ 8 h after administration. There was no significant difference in the whole GI luminal pH between the untreated control rats and the lansoprazole-pretreated rats (Figure 4A,B). 

## 4. Discussion

It is generally accepted that unformulated pure 5-ASA is rapidly and extensively absorbed in the proximal intestine. As described in the Introduction, it has been reported that 91% of the total 5-ASA dose (20 mg) was absorbed over 24 h from the proximal small intestine in rats and more than 99% of the total 5-ASA dose (150 mg as a solution) was absorbed in the human proximal intestine [9,10]. In those studies; however, 5-ASA was administered into the duodenum of the closed small intestinal loop and retained there for more than several hours. In an in vivo situation, orally administered drugs traverse the whole digestive tract, including the stomach, small intestine, large intestine, rectum, and anus, and are exposed to various luminal fluids, ingredients including food digestives, and intestinal microflora with different pHs during their passage. In addition, a high once-daily oral dose is used in clinical practice, although 5-ASA is a BCS Class IV drug with low solubility and permeability [12,14,15,16,17,18,19]. Accordingly, it was suspected that 5-ASA absorption in the proximal intestine may be limited due to the low solubility and membrane permeability, especially at a high oral dose of 5-ASA, and the remaining unabsorbed 5-ASA in the small intestine would be delivered to the large intestine without specific delivery systems. In this study, the 5-ASA and AC-5-ASA concentrations in the intestinal membrane were determined at 3 and 8 h after the oral administration. 5-ASA is the active ingredient that reflects the treatment efficacy for UC. AC-5-ASA is a metabolite of 5-ASA produced by N-acetyltransferase (NAT)-mediated metabolism, and it has been reported that AC-5-ASA may be a useful 5-ASA efficacy biomarker [26]. Figure 2 and Figure 3 show that some fraction of the orally administered 5-ASA reached the cecum/proximal colon within 3 h. Then, the remaining unabsorbed 5-ASA in the small intestine reached the distal intestine within 8 h after the administration. In this section, we discuss the factors that influenced the 5-ASA dissolution amounts in vivo, such as the luminal pH, water volume, and 5-ASA regional membrane distribution along the digestive tract, to examine whether unformulated 5-ASA was efficiently delivered to the colonic region at a high oral dose of 5-ASA. 

### 4.1. Factors That Affected the 5-ASA Dissolution Amount In Vivo (Luminal pH, Water Volume) 

5-ASA is a zwitterionic compound with a pKa_1_ = 2.30 (or 3.0) for the carboxyl group, a pKa_2_ = 5.69 (or 6.0) for the amino group, and a pKa_3_ = 13.9 for the phenol group [10,27,28,29]. Dissociation of the phenol group is negligible in the physiological pH range (approximately pH 1–8). Figure 1 shows that the 5-ASA solubility is higher at pH < 2 (most positively charged) and pH > 7 (most negatively charged) compared with the zwitterionic pH range (pH 4, 5, and 6.5). It has been reported that 5-ASA solubility is pH-dependent, where the solubility at pH 1.2 > solubility at pH 4.5 < solubility at pH 6.8 [15]. Furthermore, the pH-dependent solubility of 5-ASA has been reported as follows: 0.5% at pH 1, 0.1% at pHs 2–5, 0.2% at pH 6, 0.5% at pH 7, 1.0% at pH 8, and 3.3% at pH 9 [30]. 

The dissolved amounts of 5-ASA in each gastrointestinal tract region in vivo depend on the regional pH, buffer capacity, and luminal water volume. The regional pH of luminal fluids reported in rats and humans was as follows: in rats, pH 4.16 ± 0.01 in the stomach, pH 5.99 ± 0.01 in the proximal small intestine, pH 6.34 ± 0.01 in the mid intestine, pH 6.76 ± 0.04 in the distal small intestine, pH 6.08 ± 0.01 in the cecum, and pH 5.89 ± 0.01 in the proximal colon; in humans, pH 1.0–2.5 in the stomach, pH 3.1–6.7 in the proximal intestine, pH 7.1 ± 0.5 in the mid-small intestine, and pH 7.2 ± 0.9 (pH 6.0–7.7) in the distal small intestine [31]. Similarly, the intestinal luminal pH in healthy humans has been reported as follows: pH 6.25 ± 0.13 in the duodenum, pH 6.95 ± 0.12 in the jejunum, and pH 7.64 ± 0.16 in the ileum during the digestive period of a semiliquid 300 mL test meal, and pH 6.31 ± 0.18 in the duodenum, 7.08 ± 0.18 in the jejunum, and 7.81 ± 0.12 in the ileum during the subsequent inter-digestive period [29]. The small-intestinal luminal pH determined using a radiotelemetry capsule in healthy volunteers was as follows: pH 5.5–7.0 (mostly pH 6.4–6.8) in the proximal small intestine, pH 6.5–7.7 (mostly pH 7.3–7.7) in the distal small intestine, pH 5.5–7.5 (mostly pH 5.8–6.8) in the cecum/right colon, and pH 6.5–7.5 (mostly pH 7.0–7.2) in the left colon [32]. Similarly, the average pHs of the gastrointestinal fluid in healthy humans were pH 1.82, 4.97, 5.67, 6.17, and 6.62 in the stomach, duodenum, proximal jejunum, middle jejunum, and distal jejunum, respectively [33]. The luminal pH, especially stomach and proximal small intestine in humans, is slightly altered by food intake as follows: in a fasted condition, pH 2.9 ± 1.97 in the stomach, pH 6.71 ± 0.44 in the duodenum, pH 6.8 ± 0.4 in the jejunum, and pH 6.5 ± 0.2 in the ileum; in a fed condition, pH 4.3–5.4 in the stomach, pH range 3.1–6.0 in the duodenum, pH range 5.2–6.0 in the jejunum, and pH range 6.8–8.0 in the ileum [14]. The luminal pH of fed rats observed in the present study (Figure 2C,D and Figure 4A,B) was close to that of fed conditions in humans [14]. It was found that the dosing vehicle pH, the 5-ASA solubility in the dosing preparations, and the treatment of rats with lansoprazole seemed to not affect the luminal pH much, and therefore, the 5-ASA solubility in vivo in fed rats with food in their stomachs and intestinal tracts. Thus, the 5-ASA solubility is thought to be determined by the intrinsic regional pH rather than the dosing vehicle solubility.

It has been reported that the solubility of 5-ASA, an ionized drug, correlates positively with the gastrointestinal pH in three animal species (rats, rabbits, and pigs) and is significantly influenced by the pH difference. In contrast, the solubility of prednisolone, an un-ionizable drug, is not correlated significantly with pH changes, buffer capacity, osmolality, or surface tension [31]. Regarding the buffer capacity of luminal fluids, foods can cause resistance to a pH change due to the presence of acid/base groups. In addition, dynamic pH changes occur in human gastrointestinal fluids due to the low buffer capacity along the gastrointestinal tract in fasted and fed states in healthy subjects [34,35]. In humans, 5-ASA solubility increases down the gastrointestinal tract as follows: 1.97 ± 0.25, 3.26 ± 0.08, 6.24 ± 1.13, and 7.95 ± 0.21 mg/mL in the jejunal, ileal, ascending, and transverse/descending colonic fluids, respectively. 5-ASA solubility depends on the pH and buffer capacity (the ability of a solution to resist changes in pH), in which the buffer capacity was more important than the pH [36]. The lesser effect of dosing vehicle and lansoprazole treatment of rats on the luminal fluid pH in the present study (Figure 2C,D and Figure 4A,B) may have been due to the food intake of the rats employed (fed rats).

To estimate the 5-ASA solubility in luminal fluid in vivo, the water volumes in the gastrointestinal tract are an important factor, in addition to the luminal pH values. The reported water volumes were 0.98 ± 0.4 and 0.81 ± 1.3 mL in the fed and fasted mice, respectively, and 7.8 ± 1.5 and 3.2 ± 1.8 mL in the fed and fasted rats, respectively. It has been reported that mice and rats have a higher water volume per body weight than humans [37]. In humans, it has been reported that the fasted stomach contains 35 ± 7 mL of resting water, and upon drinking 240 mL water, the gastric fluid rose to 242 ± 9 mL and returned to the baseline 45 min after the drink. The fasted small bowel contained a total resting water volume of 43 ± 14 mL, which increased to a maximum of 94 ± 24 mL distributed within 15 ± 2 pockets of 6 ± 2 mL each 12 min after water ingestion. When the gastric water volume returned to the baseline 45 min after water ingestion, the total intestinal water volume was 77 ± 15 mL distributed into 16 ± 3 pockets of 5 ± 1 mL each [14]. In fasted human subjects, the mean resting volume of gastric water was 25 ± 18 mL (*n* = 120). When fasted humans ingested 240 mL of water, 85 ± 13% (*n* = 22) of the initially available gastric volume (resting volume plus 240 mL) was emptied after 30 min [38]. In addition, it has been reported that the fasted ascending, transverse, and descending colon contains 11 ± 5 pockets of resting liquid with a total volume of 2 ± 1 mL (average) in humans. After the 240 mL water drink, the colonic fluid increased to 7 ± 4 mL after 30 min, with a high interindividual variability. The number of colonic fluid pockets ranged from 0 to 89 and the total colonic freely mobile fluid volume ranged from 0 to 49 mL among the subjects [39]. In addition, it has been reported that the fluid volume estimated using magnetic resonance imaging (MRI) over 30 min was 275 to 46.5 mL in the stomach, 5.6 to 20.4 mL in the proximal small intestine, 36.4 to 44.1 mL in the distal small intestine, and 42 to 64.5 mL in the total small intestine 120 min after ingesting 240 mL of water [33].

The extent of 5-ASA dissolution is determined by the regional luminal pH, buffer capacity, and regional water volume, where the dissolved 5-ASA is thought to be readily absorbed from the luminal fluid. As described in the Introduction, it has been reported that 91% of the total 5-ASA dose was absorbed in the proximal small intestine when rats received 20 mg of 5-ASA via a duodenostomy tube. In that loop study, the ileostomy effluents, bile, blood, and urine were collected for 24 h after administration: about 6% of the administered dose was excreted in bile, 61% in the urine, and 9% in the ileostomy effluent. In these biological samples, most were N-acetyl-5-aminosalycilic acid (AC-5-ASA), an acetylated 5-ASA metabolite [9]. Similarly, when human volunteers received 150 mg pure 5-ASA in a 100 mL pH 7.0 buffer solution as an installation into the 100 cm long proximal part of the small bowel, only 0.1% of the total 5-ASA dose was recovered in the duodenal aspirate [10]. Thus, dissolved 5-ASA exhibited high absorption. However, 5-ASA is accepted as being a low-solubility, low-permeability, and high-dose drug. It has been reported that high oral 5-ASA doses (2 to 4 g/day) are more effective than lower doses in the treatment of patients with mild-to-moderate active UC. High 5-ASA doses (4 g/day) have also been reported as effective in CD treatment, predominantly in patients with ileitis [40]. In addition, clinical trials demonstrated that a high-dose, once-daily formulation of 5-ASA (MMX mesalamine, Lialda) at 2.4 or 4.8 g/day was superior to a placebo in inducing remission in active mild-to-moderate UC. With a high-dose formulation of 1.2 g 5-ASA per tablet, MMX mesalamine could be administered conveniently using two to four pills, once a day [4]. 

The following data were adopted to estimate the 5-ASA dissolution amount in the human small intestine: the stomach was pH 4.3–5.4 after the meal, and 5-ASA preparations were usually ingested after the meal [14]. Given the 5-ASA solubility in the stomach after the meal, 2.0 mg/mL (the reported datum was 1.97 ± 0.25 mg/mL) of 5-ASA was adopted, in which the solubility in the stomach was assumed to be the same as that in the jejunum with pH 4.3–5.4 [36]. The maximal water volume in the stomach after the ingestion of 240 mL water was assumed to be 280 mL (the reported datum was 275 mL) [33]. 5-ASA solubility in the small intestine was adopted as 3.5 mg/mL (the reported datum was 3.26 ± 0.08 mg/mL in the ileum) and the water volume was assumed to be 100 mL (the maximum reported datum was 94 ± 24 mL) [14], in which the solubility in the ileum was used because the ileum has a higher luminal pH, and thus, exhibits higher 5-ASA solubility. The estimated maximum 5-ASA solubility was 560 mg (=2.0 g/mL × 280 mL) in the stomach and 350 mg (=3.5 mg/mL × 100 mL) in the whole small intestine, and the total solubility was 910 mg. The estimated value of 910 mg in the human small intestine, including the stomach, would be overestimated because the 5-ASA suspension will not be dissolved immediately when exposed to different pHs and ingested water rapidly leaves the gastrointestinal tract. At the higher oral dose of 5-ASA, the 5-ASA dissolved fraction in the gastro/small intestinal tract was considered to be limited, and the remaining un-dissolved and unabsorbed 5-ASA in the small intestine was delivered to the colonic region, as observed in the present study (Figure 3 and Figure 4).

### 4.2. 5-ASA Membrane Distribution Along the Digestive Tract

The present study’s objective was to clarify whether unformulated pure 5-ASA is efficiently delivered to the colonic region when a high oral dose of 5-ASA is ingested. To examine this, the 5-ASA and AC-5-ASA membrane distributions along the digestive tract after 5-ASA oral administration as a suspension was measured in rats in vivo. In a clinical study, a high-dose, once-daily formulation of 5-ASA (such as 2.4 or 4.8 g/day) with multi-matrix system (MMX) technology has been reported as superior to placebo at inducing the remission of active mild-to-moderate UC [4,41]. For example, MMX mesalazine (5-ASA, once-daily 4.8 g/day) reportedly showed a higher efficacy with no difference in safety in mildly to moderately active UC compared with the pH-dependent release of 5-ASA at 3.6 g/day [42]. The 5-ASA concentration in the membrane is considered to be determined by the 5-ASA solubility in the regional luminal fluid, the transporter-mediated influx transport rate from the luminal fluid into the membrane, the transfer rate from the membrane into the portal vein, and the metabolic rate of 5-ASA by NAT in the membrane. The 5-ASA influx transporter polyspecific carnitine/organic cation transporter (OCTN1/SLC22A4) is involved, at least in part, in the 5-ASA gastrointestinal absorption [43]. Moreover, polypeptide 2B1 (OATP2B1) and sodium-coupled monocarboxylate transporter 1 (SMCT1) have been reported to be mediated in the 5-ASA uptake, with a greater contribution from OATP2B1 than SMCT1 [24]. 5-ASA has Ac-CoA-dependent metabolism; is saturable with a Km value of 5.8 μM and is inhibited by alternative NAT substrates, such as isoniazid and p-aminobenzoylglutamate [44]. The membrane concentration of AC-5-ASA, a main 5-ASA metabolite, is determined by the metabolic rate of 5-ASA by NAT in the membrane, the transfer rate into the portal vein from the membrane, and the efflux rate by MRP2 from the membrane into the luminal lumen. It has been reported that the variation in 5-ASA’s oral bioavailability between individuals is mostly due to the interindividual variation in NAT activities along the gastrointestinal tract [11,16,45]. Although AC-5-ASA is a substrate for MRP2, 5-ASA is not a substrate for the ATP-dependent efflux transporters P-glycoprotein and MRP 2. Regarding the MRP2-mediated efflux transport of AC-5-ASA in Caco-2 cells, it has been reported that the PappBA/PappAB (apical-to-basolateral permeability/basolateral-to-apical apparent permeability) ratio of AC-5-ASA was 4.89, and quercetin and MK571 (both are MRP inhibitors) inhibited the permeability ratio of AC-5-ASA to almost 1 [22,23,24]. 

In this study, 5-ASA membrane distribution was evaluated using the total 5-ASA and AC-5-ASA concentrations in the plasma, urine, and intestinal membrane after the administration. Rapid absorption of 5-ASA after administration was observed, in which the plasma and urine total concentrations were higher at 0.5 h after administration (Table 2). At 3 h, the small intestine showed higher total (5-ASA + AC-5-ASA) concentrations, especially in the ileum region, than the large intestine. There was no significant difference in the total 5-ASA and AC-5-ASA plasma, urine, and membrane concentrations and the regional luminal pH along the gastrointestinal tract at 0.5 and 3 h after the administration between the 5-ASA suspension in water and the solution in 5% NaHCO_3_ (Table 2, Figure 2A–D). These results suggest that the dissolved 5-ASA fraction in the small intestine could be rapidly absorbed in the small intestine; however, the amount of 5-ASA absorbed was limited, and the 5-ASA solubility in vivo possibly depended on the intrinsic luminal pH and food ingredients. The 5-ASA solubility in the dosing preparation (suspension in water vs. solution in 5% NaHCO_3_) and the preparation pH did not affect the 5-ASA uptake transport (absorption/distribution). The similar 5-ASA membrane distributions between the two different preparations (suspension and solution) could have been due to the similar 5-ASA water solubilities in the presence of food in the gastrointestinal tract, where foods are known to cause resistance to pH change due to the presence of acid/base groups [34,35]. 5-ASA saturable membrane transport via influx transporters may have also contributed to the 5-ASA absorption/distribution in the small intestine. Such similar 5-ASA distributions in the large intestinal membrane and regional luminal pHs after oral administration of 5-ASA were also observed at 8 h for four different dosing preparations: suspension in water, 0.1 M HCl, or 0.1 M NaOH in the untreated control rats and the solution in 5% NaHCO_3_ in the lansoprazole-pretreated rats (Figure 3 and Figure 4). In a clinical setting, 5-ASA medicines are usually taken after meals. In such cases, the changes in the luminal fluid pH and solubility of 5-ASA may be small, if any, because the increase in 5-ASA solubility only occurs at pH < 7 (Figure 1), and such changes in the luminal fluid pH are thought to be rare or non-existent in in vivo conditions. 

## 5. Conclusions

In this study, it was speculated that the dissolved 5-ASA fraction was absorbed in the proximal small intestine rapidly; however, the extent of 5-ASA absorption in the proximal small intestine was limited due to poor solubility, saturable membrane permeability, and a high dose. The higher the 5-ASA dose, the more 5-ASA that was delivered to the colonic region without specific delivery preparation.

## Figures and Tables

**Table 1:** Solubility and the dissolved and solid fractions of 5-ASA in the dosing vehicle for the rat membrane distribution studies.

Vehicle	Solubility (mg/mL)	Dissolved Fraction (%)	Solid Fraction (%)
Water	1.6	11.7	88.3
0.1 M HCl	3.9	28.6	71.4
0.1 M NaOH	13.1	96.1	3.9
5% NaHCO_3_	58.3	100	0

5-ASA was administered orally as a suspension (water, 0.1 M HCl, and 0.1 M NaOH) or solution (5% NaHCO_3_) at a 30 mg/2.2 mL/kg dose to rats.

**Table 2:** Plasma concentrations, urinary excretions, and membrane concentrations of 5-ASA and AC-5-ASA after oral administration of 5-ASA suspension in water to untreated control rats and 5-ASA solution in 5% NaHCO_3_ to lansoprazole-pretreated rats at a 30 mg/2.2 mL/kg dose.

	5-ASA Suspension in Water			5-ASA Solution in 5%NaHCO_3_		
	(Untreated Control Rats)			(Lansoprazole-Treated Rats)		
	5-ASA	AC-5-ASA	Total	5-ASA	AC-5-ASA	Total
Concentration in plasma (µg/mL)						
At 0.5 h	5.3 ± 4.3	12.0 ± 3.1	17.2 ± 7.2	6.2 ± 2.9	9.4 ± 1.9	15.6 ± 4.8
At 3 h	0.3 ± 0.0	1.9 ± 0.7	2.1 ± 0.8	0.4 ± 0.1	2.0 ± 0.4	2.4 ± 0.4
At 8 h	0.3 ± 0.3	0.7 ± 0.2	1.0 ± 0.1	0.5 ± 0.0	0.8 ± 0.4	1.3 ± 0.4
Concentration in urine (µg/mL)						
At 0.5 h	40.6 ± 28.6	1028.5 ± 642.7	1069.1 ± 669.9	21.3 ± 6.2	824.9 ± 196.5	846.2 ± 195.7
At 3 h	4.8 ± 2.8	443.6 ± 0.7	448.3 ± 169.5	8.2 ± 7.5	522.8 ± 86.9	531.0 ± 86.0
At 8 h	6.6 ± 6.7	320.4 ± 174.3	327.0 ± 180.8	3.2 ± 0.7	320.1 ± 66.9	323.4 ± 67.5
Concentration in GI membrane at 3 h (µg/g)						
Small intestine	17.5 ± 3.6	69.1 ± 32.1	86.6 ± 31.5	19.4 ± 2.5	38.7 ± 9.2	58.1 ± 9.9
Large intestine	6.8 ± 6.1	19.8 ± 13.3	26.6 ± 19.3	7.8 ± 5.0	16.7 ± 6.8	24.5 ± 10.0
Whole GI tract	24.3 ± 9.7	89.0 ± 26.9	113.2 ± 29.5	27.2 ± 6.4	55.4 ± 14.5	82.1 ± 18.8
Concentration in GI membrane at 8 h (µg/g)						
Small intestine	9.7 ± 2.2	9.2 ± 1.6	18.9 ± 2.3	12.1 ± 4.8	19.8 ± 14.9	31.9 ± 19.4
Large intestine	10.1 ± 3.1	40.7 ± 18.8	50.9 ± 21.9	10.7 ± 0.9	63.6 ± 30.4	74.3 ± 31.3
Whole GI tract	19.9 ± 3.9	49.9 ± 17.5	64.1 ± 20.3	22.9 ± 5.6	83.4 ± 41.1	106.3 ± 46.6

GI: gastrointestinal. 5-ASA, 5-aminosalycilic acid; AC-5-ASA, N-acetyl-5-ASA. Each value represents the mean ± S.D. of three trials. There was no significant difference in each corresponding value between the untreated control and lansoprazole-treated rats including the membrane concentrations in the small and large intestines at 3 h and 8 h.

## Data Availability

The data presented in this study are available on request from the corresponding author due to ethical reasons (Animal Welfare).

## Supplementary Materials

The following supporting information can be downloaded at: https://www.mdpi.com/article/10.3390/pharmaceutics16121567/s1, Figure S1: Spectrum of ^1^H-NMR of AC-5-ASA (A), Prop-5-ASA (B), and Prop-4-ASA (C).

## References

[1] Rubin D.C., Shaker A., Levin M.S. (2012). Chronic intestinal inflammation: Inflammatory bowel disease and colitis-associated colon cancer. Front. Immunol..

[2] Seyedian S.S., Nokhostin F., Malamir M.D. (2019). A review of the diagnosis, prevention, and treatment methods of inflammatory bowel disease. J. Med. Life.

[3] Dahiya D.S., Perisetti A., Kichloo A., Singh A., Goyal H., Rotundo L., Vennikandam M., Shaka H., Singh G., Singh J. (2022). Increasing thirty-day readmissions of Crohn’s disease and ulcerative colitis in the United States: A national dilemma. World J. Gastrointest. Pathophysiol..

[4] Hu M.Y., Peppercorn M.A. (2008). MMX mesalamine: A novel high-dose, once-daily 5-aminosalicylate formulation for the induction of remission in mild to moderate UC and has a favorable safety profile. The treatment of ulcerative colitis. Expert Opin. Pharmacother..

[5] Gisbert J.P., Gomollón F., Hinojosa J., López San Román A. (2010). Adherence of gastroenterologists to European Crohn’s and Colitis Organisation consensus on ulcerative colitis: A real-life survey in Spain. J. Crohn’s Colitis.

[6] Punchard N.A., Greenfield S.M., Thompson R.P. (1992). Mechanism of action of 5-arninosalicylic acid. Mediat. Inflamm..

[7] Greenfield S.M., Punchard N.A., Teare J.P., Thompson R.P.H. (1993). Review article: The mode of action of the aminosalicylates in inflammatory bowel disease. Aliment. Pharmacol. Ther..

[8] Miyake M., Fujishima M., Nakai D. (2017). Inhibitory Potency of Marketed Drugs for Ulcerative Colitis and Crohn’s Disease on PEPT1. Biol. Pharm. Bull..

[9] Shafii A., Chowdhury J.R., Das K.M. (1982). Absorption, enterohepatic circulation, and excretion of 5-aminosalicylic acid in rats. Am. J. Gastroenterol..

[10] Nielsen O.H., Bondensen S. (1983). Kinetics of 5-aminosalicylic acid after jejunal instillation in man. Br. J. Clin. Pharmacol..

[11] Ireland A., Priddle J.D., Jewell D.P. (1990). Acetylation of 5-aminosalicylic acid by isolated human colonic epithelial cells. Clin. Sci..

[12] Smetanová L., Stětinová V., Kholová D., Kuneš M., Nobilis M., Svoboda Z., Květina J. (2013). Transintestinal transport mechanisms of 5-aminosalicylic acid (in situ rat intestine perfusion, Caco-2 cells) and Biopharmaceutics Classification System. Gen. Physiol. Biophys..

[13] Myers B., Evans D.N., Rhodes J., Evans B.K., Hughes B.R., Lee M.G., Richens A., Richards D. (1987). Metabolism and urinary excretion of 5-amino salicylic acid in healthy volunteers when given intravenously or released for absorption at different sites in the gastrointestinal tract. Gut.

[14] Mudie D.M., Murray K., Hoad C.L., Pritchard S.E., Garnett M.C., Amidon G.L., Gowland P.A., Spiller R.C., Amidon G.E., Marciani L. (2014). Quantification of gastrointestinal liquid volumes and distribution following a 240 mL dose of water in the fasted state. Mol. Pharm..

[15] Martir J., Flanagan T., Mann J., Fotaki N. (2020). Impact of Food and Drink Administration Vehicles on Paediatric Formulation Performance: Part 1—Effects on Solubility of Poorly Soluble Drugs. AAPS Pharm. SciTech.

[16] Zhang Y., Wo S.K., Leng W., Gao F., Yan X., Zuo Z. (2022). Population pharmacokinetics and IVIVC for mesalazine enteric-coated tablets. J. Control. Release.

[17] Kamm M.A., Sandborn W.J., Gassull M., Schreiber S., Jackowski L., Butler T., Lyne A., Stephenson D., Palmen M., Joseph R.E. (2007). Once-daily, high-concentration MMX mesalamine in active ulcerative colitis. Gastroenterology.

[18] Georgaka D., Butler J., Kesisoglou F., Reppas C., Vertzoni M. (2017). Evaluation of dissolution in the lower intestine and its impact on the absorption process of high dose low solubility drugs. Mol. Pharm..

[19] Le Berre C., Roda G., Nedeljkovic Protic M., Danese S., Peyrin-Biroulet L. (2020). Modern use of 5-aminosalicylic acid compounds for ulcerative colitis. Expert Opin. Biol. Ther..

[20] Zhou S.Y., Piyapolrungroj N., Pao L., Li C., Liu G., Zimmermann E., Fleisher D. (1999). Regulation of paracellular absorption of cimetidine and 5-aminosalicylate in rat intestine. Pharm. Res..

[21] Li P., Luo J., Jiang Y., Pan X., Dong M., Chen B., Wang J., Zhou H., Jiang H., Duan Y. (2024). Downregulation of OATP2B1 by proinflammatory cytokines leads to 5-ASA hyposensitivity in Ulcerative colitis. Chem. Biol. Interact..

[22] Xin H.W., Schwab M., Klotz U. (2006). Transport studies with 5-aminosalicylate. Eur. J. Clin. Pharmacol..

[23] Yoshimura S., Kawano K., Matsumura R., Sugihara N., Furuno K. (2009). Inhibitory effect of flavonoids on the efflux of N-acetyl 5-aminosalicylic acid intracellularly formed in Caco-2 cells. J. Biomed. Biotechnol..

[24] Kamishikiryo J., Matsumura R., Takamori T., Sugihara N. (2013). Effect of quercetin on the transport of N-acetyl 5-aminosalicylic acid. J. Pharm. Pharmacol..

[25] Vos M.D., Verdievel H., Schoonjans R., Beke R., Weerdt G.A.D., Barbier F. (1991). High-performance liquid chromatographic assay for the determination of 5-aminosalicylic acid and acetyl-5-aminosalicylic acidconcentrations in endoscopic intestinal biopsy in humans. J. Chromatogr..

[26] Fukuda T., Naganuma M., Takabayashi K., Hagihara Y., Tanemoto S., Nomura E., Yoshimatsu Y., Sugimoto S., Nanki K., Mizuno S. (2020). Mucosal concentrations of N-acetyl-5-aminosalicylic acid related to endoscopic activity in ulcerative colitis patients with mesalamine. J. Gastroenterol. Hepatol..

[27] Allgayer H., Sonnenbichler J., Kruis W., Paumgartner G. (1985). Determination of the pK values of 5-aminosalicylic acid and N-acetylaminosalicylic acid and comparison of the pH dependent lipid-water partition coefficients of sulphasalazine and its metabolites. Arzneimittelforschung.

[28] French D.L., Mauger J.W. (1993). Evaluation of the physicochemical properties and dissolution characteristics of mesalamine: Relevance to controlled intestinal drug delivery. Pharm. Res..

[29] Goebell H., Klotz U., Nehlsen B., Layer P. (1993). Oroileal transit of slow release 5-aminosalicylic acid. Gut.

[30] (2024). Interview Form of PENTASA Tablets 250 mg, 500 mg. PENTASA Granules 94%. 2024 (May) Revision.

[31] Merchant H.A., Afonso-Pereira F., Rabbie S.C., Youssef S.A., Basit A.W. (2015). Gastrointestinal characterization and drug solubility determination in animals. J. Pharm. Pharmacol..

[32] Nugent S.G., Kumar D., Rampton D.S., Evans D.F. (2001). Intestinal luminal pH in inflammatory bowel disease: Possible determinants and implications for therapy with aminosalicylates and other drugs. Gut.

[33] Yu A., Baker J.R., Fioritto A.F., Wang Y., Luo R., Li S., Wen B., Bly M., Tsume Y., Koenigsknecht M.J. (2017). Measurement of in vivo gastrointestinal release and dissolution of three locally acting mesalamine formulations in regions of the human gastrointestinal tract. Mol. Pharm..

[34] Hens B., Tsume Y., Bermejo M., Paixao P., Koenigsknecht M.J., Baker J.R., Hasler W.L., Lionberger R., Fan J., Dickens J. (2017). Low Buffer Capacity and Alternating Motility along the Human Gastrointestinal Tract: Implications for in Vivo Dissolution and Absorption of Ionizable Drugs. Mol. Pharm..

[35] Mennah-Govela Y.A., Bornhorst G.M. (2021). Food buffering capacity: Quantification methods and its importance in digestion and health. Food Funct..

[36] Fadda H.M., Sousa T., Carlsson A.S., Abrahamsson B., Williams J.G., Kumar D., Basit A.W. (2010). Drug solubility in luminal fluids from different regions of the small and large intestine of humans. Mol. Pharm..

[37] McConnell E.L., Basit A.W., Murdan S. (2008). Measurements of rat and mouse gastrointestinal pH, fluid and lymphoid tissue, and implications for in-vivo experiments. J. Pharm. Pharmacol..

[38] Grimm M., Koziolek M., Kühn J.P., Weitschies W. (2018). Interindividual and intraindividual variability of fasted state gastric fluid volume and gastric emptying of water. Eur. J. Pharm. Biopharm..

[39] Murray K., Hoad C.L., Mudie D.M., Wright J., Heissam K., Abrehart N., Pritchard S.E., Al Atwah S., Gowland P.A., Garnett M.C. (2017). Magnetic resonance imaging quantification of fasted state colonic liquid pockets in healthy humans. Mol. Pharm..

[40] De Vos M. (2000). Clinical pharmacokinetics of slow release mesalazine. Clin. Pharmacokinet..

[41] Schreiber S., Kamm M.A., Lichtenstein G.R. (2008). Mesalamine with MMX technology for the treatment of ulcerative colitis. Expert Rev. Gastroenterol. Hepatol..

[42] Ogata H., Aoyama N., Mizushima S., Hagino A., Hibi T. (2017). Comparison of efficacy of ultimatrix mesalazine 4.8 g/day once-daily with other high-dose mesalazine in active ulcerative colitis: A randomized, double-blind study. Intest. Res..

[43] Shimizu T., Kijima A., Masuo Y., Ishimoto T., Sugiura T., Takahashi S., Nakamichi N., Kato Y. (2015). Gene ablation of carnitine/organic cation transporter 1 reduces gastrointestinal absorption of 5-aminosalicylate in mice. Biol. Pharm. Bull..

[44] Ramírez-Alcántara V., Montrose M.H. (2014). Acute murine colitis reduces colonic 5-aminosalicylic acid metabolism by regulation of N-acetyltransferase-2. Am. J. Physiol. Gastrointest. Liver Physiol..

[45] Hickman D., Pope J., Patil S.D., Fakis G., Smelt V., Stanley L.A., Payton M., Unadkat J.D., Sim E. (1998). Expression of arylamine N-acetyltransferase in human intestine. Gut.

