# Supplementary material for: 5-Aminosalicylic Acid Distribution into the Intestinal Membrane Along the Gastrointestinal Tract After Oral Administration in Rats
Source: Pharmaceutics. 2024 Dec 7;16(12):1567. doi: 10.3390/pharmaceutics16121567 (PMC11677752; doi:10.3390/pharmaceutics16121567)
Supplement: Supplementary file 1 [file pharmaceutics-16-01567-s001.zip › pharmaceutics-3301244-supplementary.pdf]

Additional files

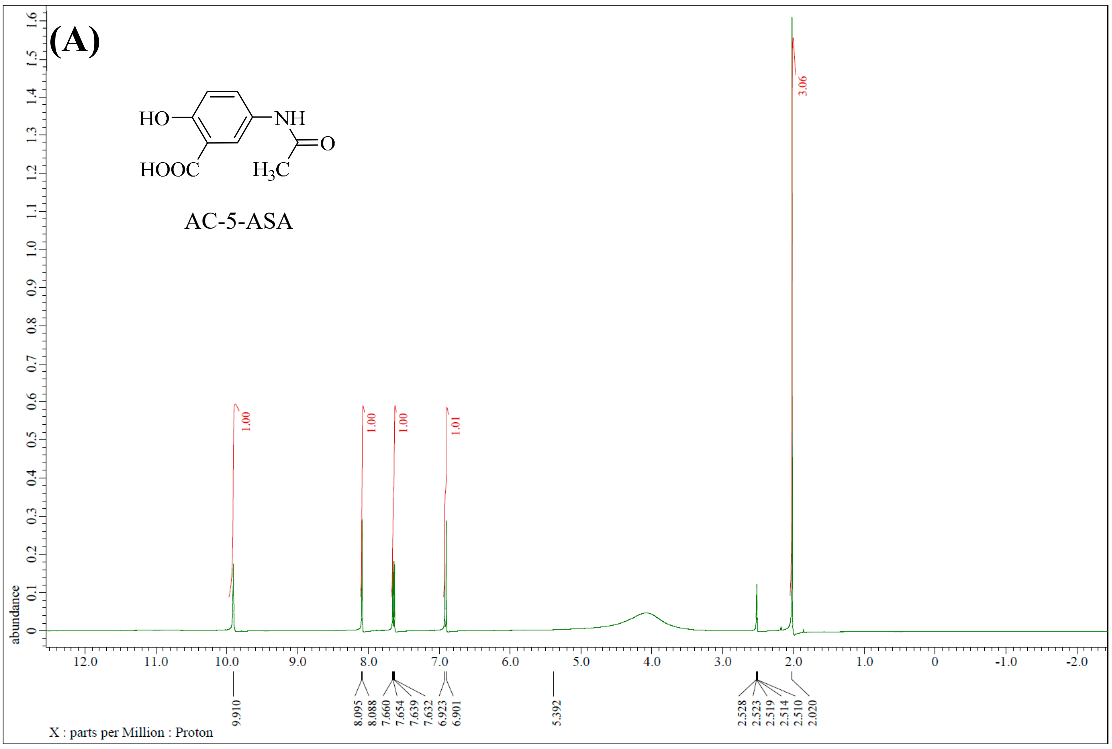

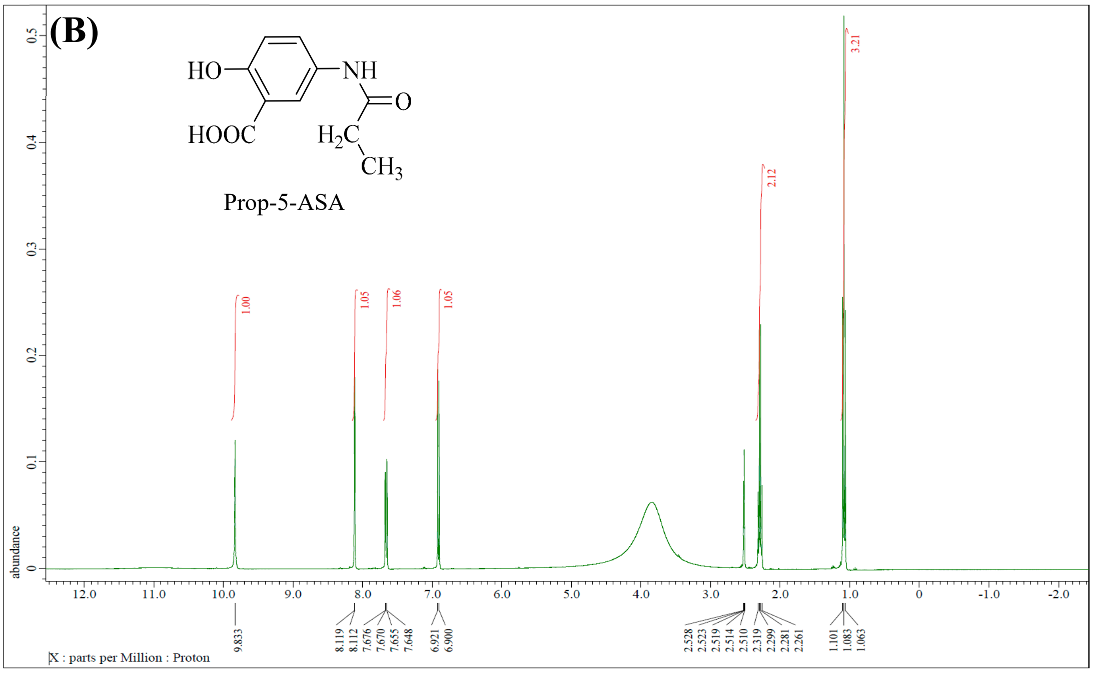

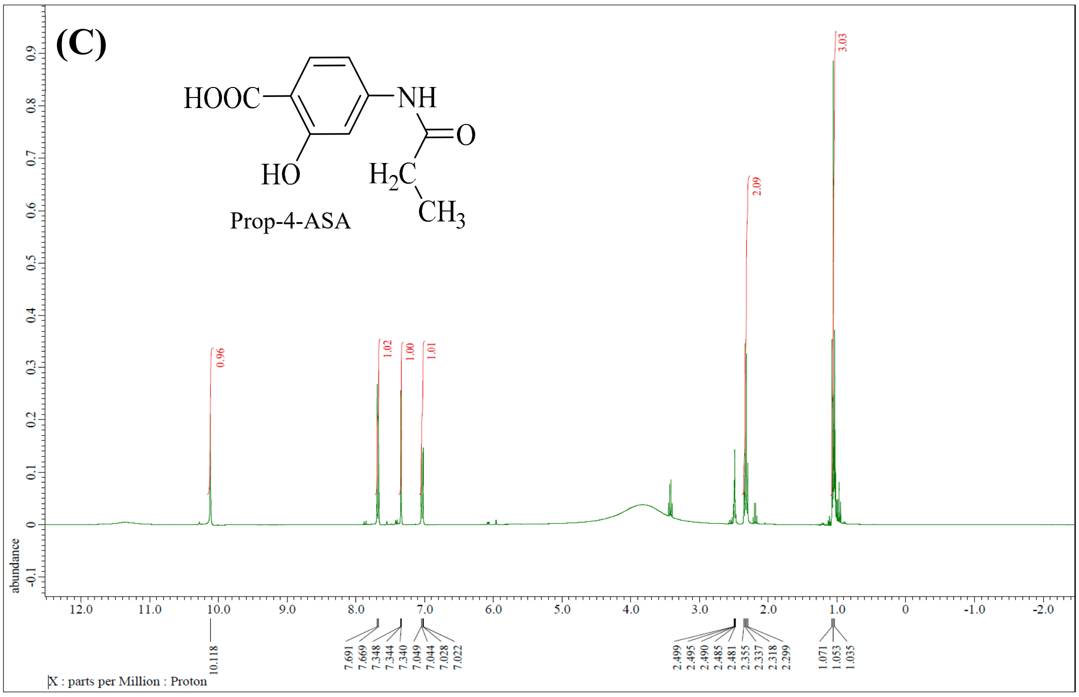

Figure S1(A), S1(B), S1(C). Proton nuclear magnetic resonance (^{1}H-NMR) spectra of AC-5-ASA (A), Prop-5-ASA (B) and Prop-4-ASA (C) recorded on a 400-MHz NMR
